# A phase II trial of recombinant MAGE-A3 protein with immunostimulant AS15 in combination with high-dose Interleukin-2 (HDIL2) induction therapy in metastatic melanoma

**DOI:** 10.1186/s12885-018-5193-9

**Published:** 2018-12-19

**Authors:** Jennifer L. McQuade, Jade Homsi, Carlos A. Torres-Cabala, Roland Bassett, Rashmi Murthy Popuri, Marihella L. James, Luis M. Vence, Wen-Jen Hwu

**Affiliations:** 1 Department of Melanoma Medical Oncology, The University of Texas MD Anderson Cancer Center, 1515 Holcombe, Unit 0430, Houston, TX 77030 USA; 2 Division of Hematology/Oncology, University of Texas Southwestern Medical Center, Dallas, TX USA; 3 Department of Pathology, The University of Texas MD Anderson Cancer Center, Houston, TX USA; 4 Department of Biostatistics, The University of Texas MD Anderson Cancer Center, Houston, TX USA; 5 Department of Immunology, The University of Texas MD Anderson Cancer Center, Houston, TX USA

**Keywords:** Melanoma, Immunotherapy, HDIL-2, MAGE-A3 CI

## Abstract

**Background:**

HDIL-2 is approved for advanced melanoma based on its durable antitumor activity. MAGE-A3 cancer immunotherapeutic (MAGE-A3 CI) is a recombinant MAGE-A3 protein combined with an immunostimulant adjuvant system and has shown antitumor activity in melanoma. We assessed the safety and anti-tumor activity of HDIL-2 combined with MAGE-A3 CI in advanced melanoma.

**Methods:**

Patients with unresectable Stage III or Stage IV MAGE-A3-positive melanoma were enrolled in this phase II study. Treatment included an induction phase of MAGE-A3 CI plus HDIL-2 for 8 cycles followed by a maintenance phase of MAGE-A3 CI monotherapy. The primary endpoints were safety and objective response assessed per RECIST v1.1. Immune biomarker and correlative studies on tumor and peripheral blood were performed.

**Results:**

Eighteen patients were enrolled. Seventeen patients were evaluable for safety and sixteen for response. Responses occurred in 4/16 (25%) patients with 3 complete responses, and stable disease in 6/16 (38%) patients with a disease control rate of 63%. The median duration of response was not reached at median follow-up of 36.8 months. Induction therapy of HDIL-2 + MAGE-A3 CI had similar toxicities to those reported with HDIL-2 alone. Maintenance MAGE-A3 monotherapy was well-tolerated. Increased immune checkpoint receptor expression by circulating T regulatory cells was associated with poor clinical outcomes; and responders tended to have increased tumor infiltrating T cells in the baseline tumor samples.

**Conclusions:**

The safety profile of HDIL-2 + MAGE-A3 CI was similar to HDIL-2 monotherapy. Maintenance MAGE-A3 CI provides robust anti-tumor activity in patients who achieved disease control with induction therapy. Immune monitoring data suggest that MAGE-A3 CI plus checkpoint inhibitors could be a promising treatment for MAGE-A3-positive melanoma.

**Trial registration:**

ClinicalTrials.gov, NCT01266603. Registered 12/24/2010, https://clinicaltrials.gov/ct2/show/NCT01266603

**Electronic supplementary material:**

The online version of this article (10.1186/s12885-018-5193-9) contains supplementary material, which is available to authorized users.

## Background

Though the prognosis for patients with metastatic melanoma has significantly improved with the FDA approval of BRAF targeted and immune therapies, [1–7] many patients will still ultimately succumb to their disease and further therapies remain urgently needed. High-dose interleukin-2 (HDIL-2) was the first FDA-approved immunotherapy for the treatment of metastatic melanoma in 1998 [8]. Though the response rates for HDIL-2 monotherapy are low (10–19% with complete response rate of 6–8%), the responses are durable [8–10].

A prior study has demonstrated that combining a peptide tumor vaccine (gp100) with HDIL-2 can improve the response rates and prolong overall survival (OS) compared to HDIL-2 monotherapy by priming the immune system to tumor-specific antigens [11]. MAGE-A3 is a tumor-specific antigen not found in normal tissues other than the testes and placenta, where the antigen expression is restricted to cells unable to present antigens to the immune system. MAGE-A3 is expressed in 60–75% of melanoma [12, 13], making this antigen a rational candidate for a vaccination approach in metastatic melanoma. The MAGE-A3 cancer immunotherapeutic (MAGE-A3 CI) is a recombinant MAGE-A3 protein combined with an immunostimulant adjuvant system (AS15). Antitumor activity of MAGE-A3 CI was demonstrated in phase II studies in multiple malignancies, including metastatic melanoma, and MAGE-A3 CI has been shown to induce both humoral and cellular immune responses against the MAGE-A3 antigen [13–15].

We hypothesized that MAGE-A3 CI combined with HDIL-2 would enhance the immune responses, thus improving the anti-tumor efficacy of HDIL-2 with acceptable tolerance. We also explored the feasibility and efficacy of maintenance MAGE-A3 CI monotherapy following the combination induction therapy. Here, we report the results of a phase II trial of MAGE-A3 CI plus HDIL-2 induction followed by maintenance MAGE-A3 CI monotherapy in patients with MAGE-A3 expressing metastatic melanoma.

## Methods

### Study participants

The eligibility criteria included age at least 18 years; histologically confirmed, stage III or IV unresectable melanoma; an Eastern Cooperative Oncology (ECOG) performance status of 0 or 1; and adequate organ function. This was a single-institution study with all patients recruited from UT MD Anderson Cancer Center. Patients were required to have available formalin-fixed paraffin-embedded (FFPE) tumor tissue for MAGE-A3 expression screening [16]. The RNA from these samples was extracted to assess the MAGE-A3 expression by quantitative PCR. The patients were considered eligible if at least one tested block was MAGE-A3-positive. Patients were additionally required to have measurable disease, defined by Response Evaluation Criteria in Solid Tumor (RECIST) v1.1 [17] and at least 1 accessible tumor for biopsy obtained within 30 days from starting treatment. Key exclusion criteria included any prior systemic therapy with the exception of interferon in the adjuvant setting, history of brain metastases, autoimmune disease and/or ANA titer > 1:80, Hepatitis B/C, or HIV, and chronic steroid use.

### Study design

Study schematic is provided as Additional file 1. In the induction phase, 300 μg of recMAGE-A3 protein (MAGE-A3) + adjuvant system 15 immunostimulant (AS-15) (see Additional file 2 for composition) were given via intramuscular injection every 2 weeks for 6 cycles and then every 3 weeks for 6 cycles. HDIL-2 was initiated on the day following MAGE-A3 CI immunization or up to eight cycles on weeks 1, 3, 9, 11, 18, 21, 27 and 30 at 720,000 IU/kg every 8 h for up to 14 doses each cycle. Following completion of the 30-week induction HDIL-2 + MAGE-A3 CI, patients who remained on study were continued on the maintenance MAGE-A3 CI alone, given every 6 weeks for 4 cycles, then every 12 weeks for 4 cycles, and then every 24 weeks for 4 cycles. Treatment was continued until disease progression, intolerable toxicity, patient withdrawal of consent, or investigator decision. Patients who permanently discontinued HDIL-2 before completing 8 cycles because of an adverse event (AE) and no signs of disease progression were allowed to proceed to maintenance phase and resume MAGE-A3 CI after resolution of the AE to grade 0 or 1. Tumor images were obtained at baseline and before every other cycle or every 8 weeks during the induction phase and then 1 week prior to each dose of maintenance MAGE-A3 CI. Assessment of antitumor activity was based on RECIST v1.1 criteria [17]. AEs were collected throughout treatment and graded according to the National Cancer Institute Common Terminology Criteria for Adverse Events, version 3.0. Attribution of AEs to study treatment was determined by the treating physician based on their assessment of exposure, time course, likely cause, and consistency with known treatment profile.

### Outcomes

The primary endpoints were overall response rate (ORR), with decision on proceeding to 2nd stage of trial based on response rate and safety profile. Tumor responses were assessed every 8 weeks during induction phase and 1 week prior to MAGE-A3 CI immunization during maintenance phase. Secondary endpoints included progression-free survival (PFS), overall survival (OS), duration of response, and biomarker correlatives.

### Molecular and immune analyses

FFPE tissue was required for MAGE-A3 expression screening, either from a fresh tumor biopsy at the time of screening or archival tissue obtained within 1 year. A baseline biopsy for gene expression profiling was performed prior to therapy initiation and optional biopsies were performed at week eight. Frozen specimens were stored in RNAlater and mRNA was extracted by standard methods [16]. Blood samples were collected at baseline and at regular intervals thereafter and peripheral blood mononuclear cells (PBMCs) isolated. MAGE-A3 gene expression with quantitative PCR (qPCR) was performed on FFPE tissue using TaqMan (Applied Biosystems, Foster City, CA) low-density arrays as previously described (see Additional file 2) [18]. Immunohistochemistry (IHC) on FFPE tumor tissue sections was performed using antibodies against PD-1, PD-L1, CD8, Granzyme B, and CD45RO (see Additional file 2). Percentage of positivity for each IHC marker was evaluated by an experienced dermatopathologist (CA Torres-Cabala). RNA from frozen baseline specimens was amplified and assayed on Affymetrix HG-U133.Plus 2.0 (Affymetrix, Santa Clara, CA) microarray gene chips by standard methods to assess gene expression of an 84-gene set previously found to be predictive of benefit in melanoma patients treated with MAGE-A3 CI [16].

Blood samples for immune biomarker studies were collected pre-MAGE-A3 dose on day 1 and at completion of HDIL-2 of each cycle in induction phase and pre-MAGE-A3 dose on day 1 of each cycle in maintenance phase. Flow cytometry was used to detect changes in immune cell populations over time and quantify MAGE-A3 specific CD8+ T cells in HLA-A 0201+ patients using an HLA-A 0201-restricted MAGE-A3 epitope.

### Statistical analysis

The study used a Two-Stage Fleming’s design, [19] with response measured by overall response (OR: complete response [CR] or partial response [PR] as defined above) in 15 patients treated in the 1st stage. The null hypothesis was that the ORR was 5%, matching the historical control rate, and the alternative hypothesis was that this could be improved to 20% with the study regimen. If none had responded, the study would have been terminated for futility. If 4 or more had responded, the study would have been terminated early for efficacy. Neither of these criteria were met, and the study was scheduled to enroll an additional 15 patients in the 2nd stage. The study was terminated for slow patient accrual after only 2 patients were enrolled in 2nd stage. Response duration was measured from the time of response until evidence of disease progression. PFS was measured from the time of treatment initiation to evidence of disease progression or death, and OS was measured from the time of treatment initiation to the time of death or last follow-up. Kaplan–Meier estimates were used to plot survival curves. Logistic regression models and Fisher’s exact test were used to assess associations of response with covariates of interest. *P*-values of < 0.05 were considered significant. R (v 3.2.2) was used for data analysis.

## Results

### Patient characteristics

Forty-four patients were screened for eligibility. Eighteen patients were eligible and enrolled between March 2011 and March 2014. Follow-up was continued until October 2017 when the last patient completed maintenance therapy. One patient withdrew consent prior to initiation of therapy. Seventeen patients were evaluable for safety and sixteen for response because one patient received one cycle of HDIL-2 without ever receiving the MAGE-A3 CI. As shown in Table 1, most patients had Stage IV disease, including 35% with M1c disease.

**Table 1:** Patient demographics and clinical characteristics

Characteristic	
Age, Mean, y (range)	55 (30–64)
Sex	
Male, No. (%)	10 (59)
Female, No. (%)	7 (41)
Stage, No. (%)	
Unresectable III	8 (47)
M1a	2 (12)
M1b	1 (6)
M1c	6 (35)
ECOG PS, No. (%)	
0	15 (88)
1	2 (12)
LDH, No. (%)	
Normal	12 (71)
>ULN	5 (29)
Mutation status	
BRAF V600E/K	12 (71)
C-KIT	1 (6)
NRAS Q61R	1 (6)
WT	3 (18)

Abbreviations: *ECOG PS* Eastern Cooperative Oncology Group performance status, *LDH* lactate dehydrogenase, *No* number, *ULN* upper limit of normal

### Safety

The adverse events were similar to those commonly seen with HDIL-2 monotherapy, including, hypotension edema (capillary leak and fluid retention), hypoalbuminemia, fatigue, fever, chills, nausea, vomiting, rash and pruritus, myalgia, arthralgia, and elevated liver enzymes (Table 2). One patient discontinued HDIL-2 after 2 cycles secondary to significant AEs (atrial fibrillation, excess fluid retention, and hypotension) but continued with maintenance MAGE-A3 CI. Few toxicities were attributed to the MAGE-A3 CI by the investigators: five patients had pain or swelling at the injection site (all Grade 1), and one patient had acute renal dysfunction with elevated creatinine (Grade 3). The patient with acute renal dysfunction discontinued MAGE-A3 CI after 2 cycles and continued HDIL-2 off-study without recurrent Grade 3 renal dysfunction.

**Table 2:** Adverse events

Event	Grade 3/4 (n)	%	Total (n)	**%**
Any event	13	76%	17	100%
Anemia	4	24%	14	82%
Anorexia	0	0%	6	35%
Arthralgia	1	6%	7	41%
Chills	0	0%	10	59%
Constipation	0	0%	4	24%
Cough	0	0%	3	18%
Decreased urine output	1	6%	1	6%
Diarrhea	1	6%	7	41%
Dry skin	0	0%	15	88%
Dyspnea	0	0%	3	18%
Edema	0	0%	14	82%
Fatigue	0	0%	14	82%
Fever	0	0%	5	29%
Headache	0	0%	7	41%
Hyperglycemia	1	6%	6	35%
Hyperkalemia	1	6%	4	24%
Hypermagnesiumia	1	6%	1	6%
Hypertension	1	6%	2	12%
Hyperuricemia	2	12%	4	24%
Hypoalbuminemia	8	47%	14	82%
Hypoglycemia	0	0%	5	29%
Hyponatremia	1	6%	2	12%
Hypophosphatemia	0	0%	3	18%
Hypotension	0	0%	7	41%
Increased alanine aminotransferase	0	0%	3	18%
Increased alkaline phosphatase	0	0%	3	18%
Increased aspartate aminotransferase	0	0%	5	29%
Increased bilirubin	2	12%	6	35%
Increased Creatinine	2	12%	6	35%
Injection site reaction	0	0%	5	29%
Lymphopenia	3	18%	4	24%
Myalgia	0	0%	10	59%
Nausea	0	0%	11	65%
Pain	1	6%	12	71%
Peripheral sensory neuropathy	0	0%	7	41%
Pruritus	1	6%	12	71%
Rash	1	6%	8	47%
Thrombocytopenia	1	6%	2	12%
Tinnitus	0	0%	3	18%
Ventricular tachycardia	1	6%	2	12%
Vomiting	0	0%	7	41%
Weight loss	0	0%	3	18%

Events occurring in > 15% of patients and all Grade 3/4 adverse events

### Efficacy

**Fig. 1:**
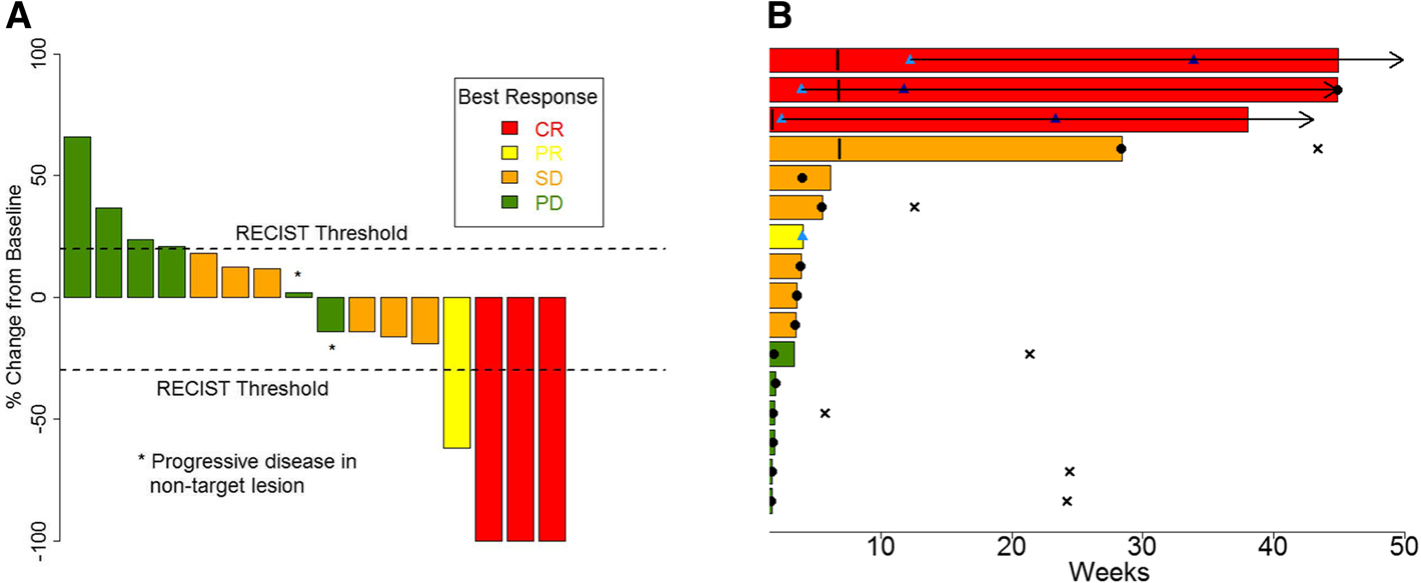
Clinical Response. **a** Waterfall plot indicating best RECIST 1.1 response as % change in lesion size from baseline. Red = complete response (CR). Yellow = partial response (PR). Orange = stable disease (SD). Green = progressive disease (PD). **b** Swimmer’s plot indicating duration of response. Red = complete response (CR). Yellow = partial response (PR). Orange = stable disease (SD). Green = progressive disease (PD). Vertical line = end of HDIL-2 + MAGE-A3 CI induction phase and beginning of maintenance phase. Blue triangle = time of partial response. Black triangle = time of complete response. Black circle = disease progression. Horizontal arrow = duration of response. X = death

Patients completed a mean of four cycles of induction therapy cycles of combination HDIL-2 and MAGE-A3 CI. Four patients continued to maintenance MAGE-A3 monotherapy. The overall response rate was 25% (3 patients with a CR and 1 patient with PR) (Fig. 1). The median time to response was 5.7 months (range 2.4 to 12.2 months). One patient (6%) achieved a PR after 8 weeks induction therapy. Three patients achieved a CR while receiving maintenance MAGE-A3 monotherapy at 11.7, 23.3, and 33.9 months respectively. Two of these patients completed all 8 cycles of HD-IL2, while the third patient only completed 2 cycles of HD-IL2 secondary to toxicity prior to proceed to maintenance MAGE-A3 CI monotherapy. The patient with a PR during the induction phase was converted to a CR with surgical resection of residual disease. With a median follow-up of 36.8 months, the median duration of response was not reached with an ongoing response up to 57.1 months. An additional six patients had stable disease (SD); thus, the overall disease control rate was 63%. The duration of SD was 2.5, 3.2, 3.3, 4.5, 5.5, and 27.5 months respectively. Median PFS was 3.6 months (95% CI 1.8 - NR), and median OS was not reached.

### Gene expression and immune profiling studies

**Fig. 2:**
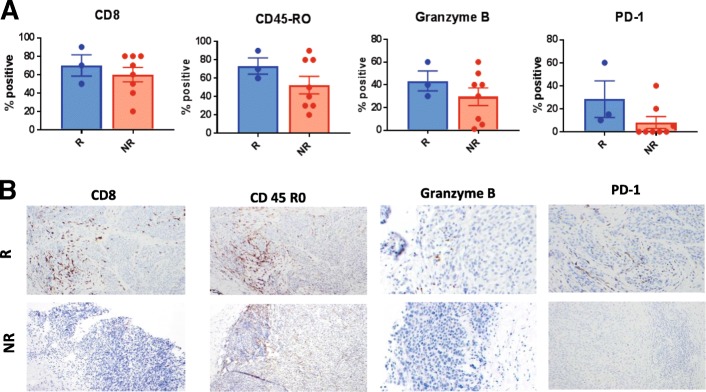
Baseline immune infiltrates in responders and non-responders. Eleven patients had baseline specimens available for immunohistochemistry. **a** Percentage of positive CD8, CD45-RO, Granzyme B, and PD-1 TILs in tumor specimens corresponding to responders (R) and non-responders (NR) patients. **b** Representative images of tissue sections from responders (R) and non-responders (NR) patients showing subsets of TILs expressing CD8, CD-45RO, PD-1 (× 100) and Granzyme B (× 200) (immunohistochemistry)

Ten patients had baseline biopsies with sufficient samples for gene expression profiling. An 84-gene signature was positive in 8 of 10 specimens (80%). Two of two patients (100%) with clinical response had a positive gene signature as did 6 of 8 patients (75%) who did not respond. Eleven baseline biopsies were analyzed by IHC to quantify immune infiltrates. PD-L1 expression was not predictive of response. Baseline tumor specimens from responders tended to have increased CD8+, CD45RO+, Granzyme B+ and PD-1+ tumor infiltrating lymphocytes though there were no statistically significant differences between responders (CR + PR) and non-responders (progressive disease, PD) (Fig. 2).

### Flow cytometry studies

**Fig. 3:**
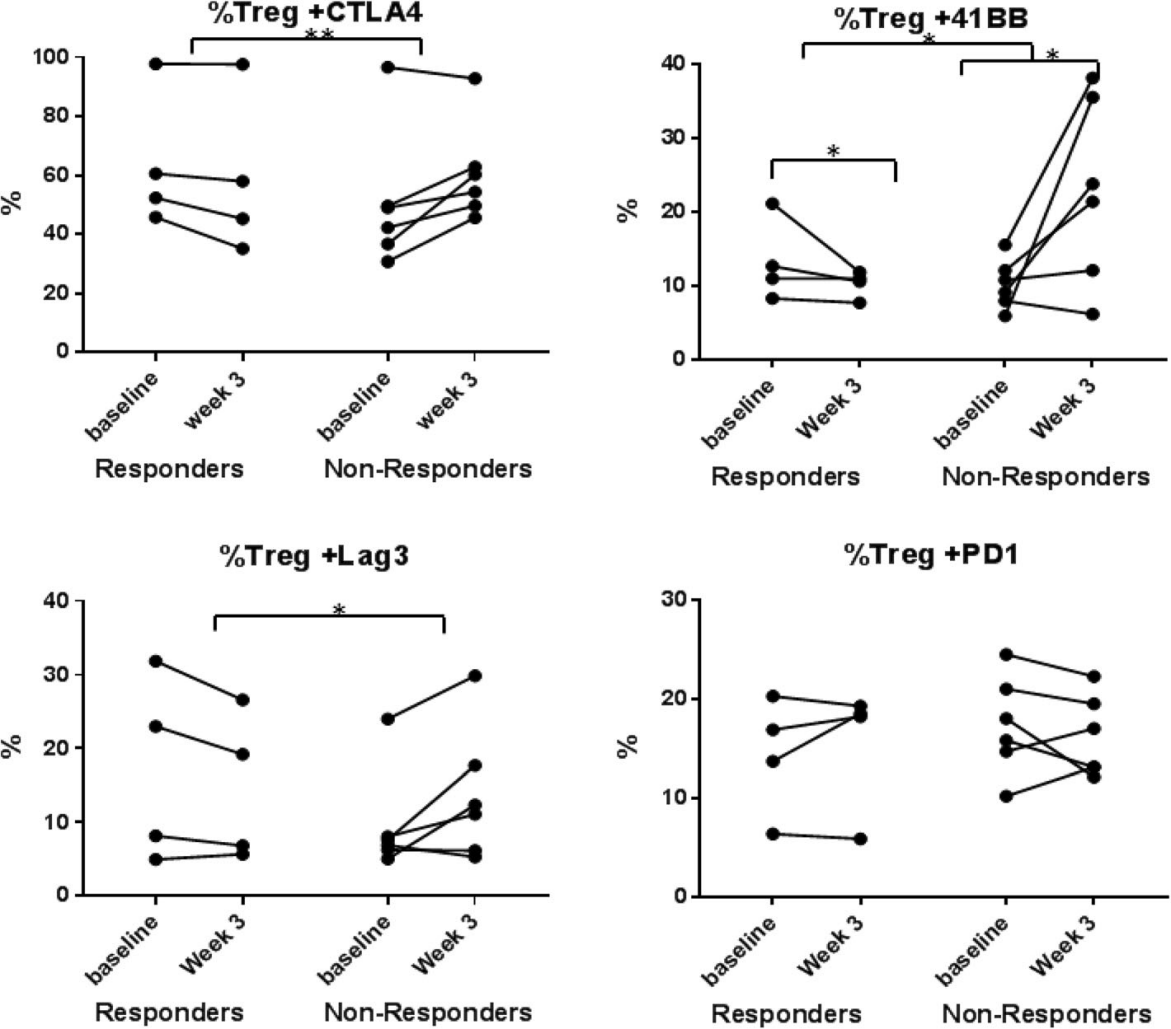
Changes in immune checkpoint expression of peripheral blood regulatory T cells with therapy. Compared to responders, non-responders show a relative increase in the expression of multiple immune checkpoint molecules on regulatory T cells on treatment including [41BB (*p* = 0.04), CTLA4 (*p* = 0.01), and LAG3 (*p* = 0.02)]

**Fig. 4:**
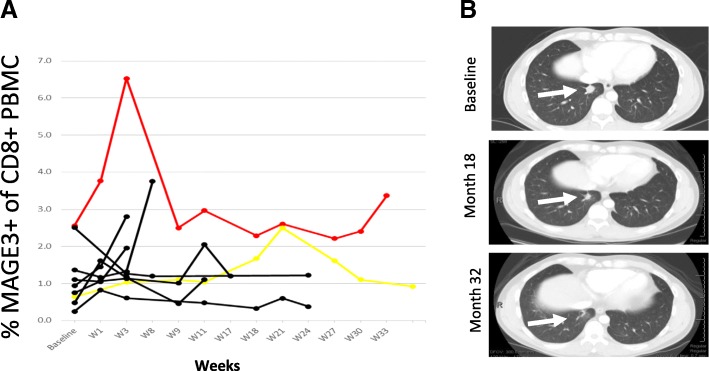
MAGE-A3 positive peripheral CD8 cells and tumor response. **a** Peripheral blood flow cytometry of MAGE-A3+ CD8 T cells over time in 9 HLA-A2.1+ patients using an HLA-A2.1-restricted MAGE-A3 epitope. Patient 16 with complete response (red) shows high MAGE-A3 + CD8 cells at baseline and a robust increase in MAGE-A3 + CD8 cells early on treatment. Non-responding patients shown in black and patient with prolonged stable disease in yellow. **b**. Cross-sectional CT images of patient 16 who had lung metastasis at baseline, achieved a partial response at 18 months and a complete response at 32 months. White arrow indicates lung metastasis

Flow cytometry was performed at baseline and week 3 on PBMCs of patients who had a response (CRs + PR, *n* = 4) and compared to those with primary progressive disease (PDs, *n* = 6). There were no differences in baseline cell populations. There were significant differences in the change of expression of multiple immune checkpoint proteins on the T regulatory cells from baseline to week 3 in the responding vs. non-responding patients with checkpoint protein expression increasing in the non-responding patients and decreasing in the responding patients from baseline to week 3 [41BB (*p* = 0.04), CTLA4 (*p* = 0.01), and LAG3 (*p* = 0.02)] (Fig. 3). Expression of 41BB on CD8+ T cells also exhibited an increase over time in non-responders compared to responders (p = 0.04). Flow cytometry for MAGE-A3 specific CD8+ T cells could be performed in 9 patients with appropriate HLA type. As only one patient with a clinical response had the appropriate HLA type (HLA-A0201+), statistical comparison of responders and non-responders was not feasible. However, the patient that achieved a CR had a high level of baseline MAGE-A3 specific CD8+ T cells and then had a further robust increase in MAGE-A3 specific CD8+ T cells at week 3 with much higher levels than any of the non-responding patients (Fig. 4).

## Discussion

HDIL-2 was the first cytokine immunotherapy approved in advanced melanoma based on infrequent but extremely durable responses. A previous phase III clinical trial suggested that combining a peptide vaccine gp100 with HDIL-2 could enhance the clinical and immunological responses over HDIL-2 alone by priming and inducing tumor-specific immunity [11]. Combining other therapies with HDIL-2 has always been pursued with extreme caution due to the high toxicity profile of HDIL-2. Our study showed that the combination of MAGE-A3 CI with HDIL-2 has an acceptable safety profile.

25% of the patients in this study achieved durable responses, with 19% of patients achieving complete responses, while prior studies with HDIL-2 monotherapy have reported response rates of 10–19% with complete response rates of 6–8%. This suggests that the combination may have synergistic anti-tumor activity, though this would of course require testing in a larger, randomized study. Furthermore, all CRs occurred while patients were receiving maintenance MAGE-A3 CI monotherapy which suggests that a shorter combination induction period could be attempted in future trials, though delayed responses have been seen with other immunotherapies as well, including HDIL-2, and thus it is not possible to attribute these effects to MAGE-A3 CI maintenance. The ideal number of HDIL-2 cycles needed to achieve the optimal immune response remains unanswered. This study was closed shortly after the 2nd stage was launched due to slow patient accrual. The patient accrual was limited by the requirement of normal cardiac stress and pulmonary function tests to avoid severe HDIL-2 toxicity as well as tumor screening for MAGE-A3 expression (18 out of 44 [40%] screened showed MAGE-A3 expression). Limitations of this trial include small sample size and the fact that it was a single-arm non-randomized study.

An interesting finding in our study is the increased expression of multiple checkpoint molecules including CTLA-4, LAG-3, and 41BB on the T-regulatory cells in non-responding patients. This could be an important mechanism of acquired resistance, and supports combining MAGE-A3 CI and/or cytokine therapy with immune checkpoint inhibitors, especially during maintenance MAGE-A3 CI monotherapy. Combining checkpoint inhibitors with other vaccines, including Talimogene Laherparepvec (T-VEC), has shown encouraging results and good safety [20, 21]. In distinction to T-VEC, an oncolytic immunotherapy, MAGE-A3 CI is based on a recombinant protein and thus its use is not limited to patients with injectable lesions.

Prior studies of MAGE-A3 CI had identified a gene signature predictive of response to treatment [16]. In our small study, this gene signature was not able to discriminate between responders and non-responders, in part because of the high overall rate of gene signature positivity (80% compared to 50–60% in prior studies) [16, 22]. Furthermore, the examination of MAGE-A3 specific CD8+ T cell responses was limited by the fact that only one of the responding patients had the appropriate HLA type required for this assay. Interestingly, despite this patient’s tumor having low protein and RNA MAGE-A3 expression, the fraction of MAGE-A3 specific CD8+ T cells at baseline was very high, indicating this patient’s immune system was primed to the MAGE-A3 antigen. At week 3 of therapy, this patient had a very robust increase in MAGE-A3 specific CD8+ T cells to levels 2-fold higher than that seen in any of the non-responding patients. Future studies may incorporate monitoring of antigen-specific T cells as a potential early predictor of response.

Importantly, combining MAGE-A3 CI with HDIL-2 appears safe and did not add any significant toxicity associated with HDIL-2. AEs attributed to MAGE-A3 CI are common to other injectable therapies, i.e. low-grade injection site reactions.

Unfortunately, further development of MAGE-A3 CI in melanoma has been stopped based on the negative results of the DERMA study in which MAGE-A3 CI was given as adjuvant therapy in resected Stage III melanoma [23]. However, results from our study as well as data from other vaccine/checkpoint inhibitor combinations suggest that there is a strong rationale to test combination MAGE-A3 CI and immune checkpoint inhibitors to augment T cell response [24].

## Conclusion

In conclusion, the combination of MAGE-A3 CI and HDIL-2 induction therapy followed by MAGE-A3 CI maintenance therapy was well tolerated and efficacious with disease control in > 60% of MAGE-A3 expressed melanoma and 19% of patients achieving durable complete responses. Though the sample size was small, this study demonstrated that MAGE-A3 CI maintenance immunotherapy can convert PRs and SDs achieved during induction therapy to CRs. The biomarker studies of our small number of patients suggest emerging expression of immune checkpoint receptors on the regulatory T-cells as a potential mechanism of acquired resistance. Thus, there is a strong rationale to combine MAGE-A3 CI and immune checkpoint inhibitors.

## Additional files

Additional file 1: Study schematic. (DOCX 13 kb)

Additional file 2: Supplementary materials and methods. (DOCX 55 kb)

## Abbreviations

AE: adverse event
AS-15: adjuvant system 15 immunostimulant
CR: complete response
ECOG: Eastern Cooperative Oncology
FFPE: formalin-fixed paraffin-embedded
HDIL-2: high-dose Interleukin-2
MAGE-A3 CI: MAGE-A3 cancer immunotherapeutic
ORR: overall response rate
OS: overall survival
PBMCs: peripheral blood mononuclear cells
PD: progressive disease
PFS: progression-free survival
PR: partial response
qPCR: quantitative polymerase chain reaction
RECIST: Response Evaluation Criteria in Solid Tumor
SD: stable disease
T-VEC: Talimogene Laherparepvec
