# Supplementary material for: A phase II trial of recombinant MAGE-A3 protein with immunostimulant AS15 in combination with high-dose Interleukin-2 (HDIL2) induction therapy in metastatic melanoma
Source: BMC Cancer. 2018 Dec 19;18:1274. doi: 10.1186/s12885-018-5193-9 (PMC6300080; doi:10.1186/s12885-018-5193-9)
Supplement: Supplementary file 1 — Study schematic. (DOCX 13 kb) [file 12885_2018_5193_MOESM1_ESM.docx]

**Treatment schematic**
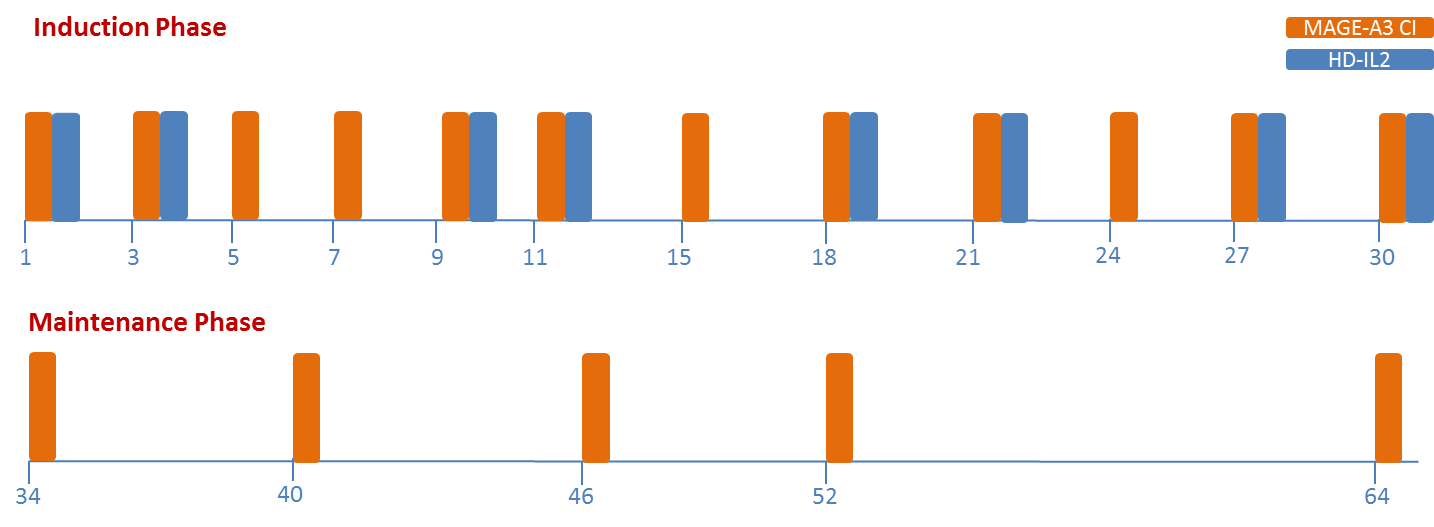


In the induction phase, 300 μg of recombinant MAGE-A3 protein (MAGE-A3) + adjuvant system 15 immunostimulant (AS-15) were given via intramuscular injection every two weeks for 6 cycles and then every 3 weeks for 6 cycles. HDIL-2 was initiated on the day following MAGE-A3 immunotherapeutic on weeks 1,3,9,11,18,21,27 and 30 at 720,000 IU/kg every 8 hours for up to 14 doses/cycle. Following completion of the 30 week induction MAGE-A3 + HDIL-2 combination, patients who remained on study were continued on the maintenance phase with MAGE-A3 immunotherapeutic alone, given every 6 weeks for 4 cycles, then every 12 weeks for 4 cycles, and then every 24 weeks for 4 cycles, up to 28 months of total therapy.
