# Supplementary material for: A phase II trial of recombinant MAGE-A3 protein with immunostimulant AS15 in combination with high-dose Interleukin-2 (HDIL2) induction therapy in metastatic melanoma
Source: BMC Cancer. 2018 Dec 19;18:1274. doi: 10.1186/s12885-018-5193-9 (PMC6300080; doi:10.1186/s12885-018-5193-9)
Supplement: Supplementary file 2 — Supplementary materials and methods. (DOCX 55 kb) [file 12885_2018_5193_MOESM2_ESM.docx]

**Supplemental Methods:**

**Mage-A3 CI composition**

The MAGE-A3 immunotherapeutic is composed of the recombinant MAGE-A3 protein administered intramuscularly with the immunostimulant AS15, a combination of 3-O-desacyl-4’- monophosphoryl lipid A (MPL, 50 µg, produced by GSK), *Quillaja saponaria* Molina, fraction 21 (QS-21, 50 µg, Licensed by GSK from Antigenics Inc, a wholly owned subsidiary of Agenus Inc., a Delaware, USA corporation) and CpG 7909 synthetic oligodeoxynucleotides containing unmethylated CpG motifs (420 µg), in a liposomal formulation. The recombinant MAGE-A3 protein was co-lyophilized with the immunostimulatory nucleotide CpG 7909 (from the AS15 immunostimulant) and reconstituted with 0·5ml of liquid adjuvant in phosphate-buffered saline.

**MAGE-A3 expression test**

*Total RNA extraction*: Manually micro-dissected tumor tissue was obtained from a deparaffinized 10-micron section of formalinfixed paraffin-embedded (FFPE) tissue. This material was used for total RNA extraction with the QIAGEN® RNeasy FFPE Kit, following manufacturer’s instructions, with addition of a DNase I oncolumn digestion step. Fifty nanograms of total RNA were used with each RT-PCR reaction.

*RT-PCR Amplification of Extracted RNA*: Each RNA sample was amplified by quantitative Reverse Transcriptase - Polymerase Chain Reaction (qRT-PCR) using Roche Molecular Diagnostics reagents (RNA Mix, Mg 2+/ Mn 2+, and Primer and Probe reagent) on a COBAS TaqMan 48 Analyzer (Roche Molecular Diagnostics). Cycling conditions were: 1 cycle of 5 min at 50°C (UNG decontamination), 1 cycle of 1 min at 95°C (denaturation), 1 cycle of 20 min at 60°C (reverse transcription), 2 cycles of 15 sec at 95°C followed by 25 sec 63°C (denaturation/ annealing), 53 cycles at 15 sec 92°C followed by 50 sec 63°C (amplification) and a final extension of 2 min 40°C. The MAGE-A3 and β-actin mRNA in the sample were co-amplified in the same reaction. Positive controls (MAGE-A3, β-actin) and negative controls (non-template, bystander) were developed for the assay and run on each PCR plate. A control to evaluate the presence of contaminating genomic DNA was also developed and run for each sample.

*Cut-off*: The cut-off was defined as 1% the relative level of MAGE-A3 to β-actin in the MZ2-MEL cell line (kind gift of the LICR Brussels). The MZ2-MEL cell line was also referred to as the GERL cell line. The outcome of a test was MAGE-A3-positive if the relative level of MAGE-A3 to β-actin in the sample was greater than or equal to 1% of the relative level of MAGE-A3 to β-actin in the GERL cell line. The cut-off was established on FFPE mouse GERL xenografts.

*Data Analysis*: Cycle threshold (Ct) values for MAGE-A3 and β-actin in a patient FFPE tumor sample were calculated using the AMPLILINK 3.1 software on the COBAS TaqMan 48 Analyzer workstation. The relative level of MAGE-A3 to β-actin in the patient tumor sample was calculated using these Ct values. If the relative level of MAGE-A3 to β-actin in the patient tumor sample was greater or equal to the cut-off of the assay, the patient was considered positive for MAGE-A3 expression and was eligible for inclusion into the trial.

**Immunohistochemistry:**

The following antibodies were used:

MAGE-A: clone 6C1, Santa Cruz Biotechnology, Santa Cruz, CA.

PD-1: clone MRQ-22,   Cell Marque, Rocklin, CA

CD45 RO: clone UCHL1, Leica Microsystems, Buffalo Grove, IL

CD8: clone C8/144B, ThermoFischer Scientific, Waltham, MA

Granzyme B: clone GrB-7, ThermoFischer Scientific, Waltham, MA
